# Inhibition of type I interferon signaling abrogates early *Mycobacterium bovis* infection

**DOI:** 10.1186/s12879-019-4654-3

**Published:** 2019-12-04

**Authors:** Jie Wang, Tariq Hussain, Kai Zhang, Yi Liao, Jiao Yao, Yinjuan Song, Naveed Sabir, Guangyu Cheng, Haodi Dong, Miaoxuan Li, Jiamin Ni, Mazhar Hussain Mangi, Deming Zhao, Xiangmei Zhou

**Affiliations:** 1 Key Laboratory of Animal Epidemiology and Zoonosis, Ministry of Agriculture, National Animal Transmissible Spongiform Encephalopathy Laboratory, College of Veterinary Medicine, China Agricultural University, Beijing, China; 2 Institute of Laboratory Animal Sciences, Chinese Academy of Medical Sciences (CAMS), Comparative Medicine Center, Peking Union Medical College (PUMC), Beijing, China; 3 School of Agriculture, Ningxia University, Ningxia, China

**Keywords:** Immunity, *Mycobacterium bovis*, Type I interferon signaling, Inflammatory response, Macrophages polarization, Neutrophils

## Abstract

**Background:**

*Mycobacterium bovis* (*M. bovis*) is the principal causative agent of bovine tuberculosis; however, it may also cause serious infection in human being. Type I IFN is a key factor in reducing viral multiplication and modulating host immune response against viral infection. However, the regulatory pathways of Type I IFN signaling during *M. bovis* infection are not yet fully explored. Here, we investigate the role of Type I IFN signaling in the pathogenesis of *M. bovis* infection in mice.

**Methods:**

C57BL/6 mice were treated with IFNAR1-blocking antibody or Isotype control 24 h before *M. bovis* infection. After 21 and 84 days of infection, mice were sacrificed and the role of Type I IFN signaling in the pathogenesis of *M. bovis* was investigated. ELISA and qRT-PCR were performed to detect the expression of Type I IFNs and related genes. Lung lesions induced by *M. bovis* were assessed by histopathological examination. Viable bacterial count was determined by CFU assay.

**Results:**

We observed an abundant expression of Type I IFNs in the serum and lung tissues of *M. bovis* infected mice. In vivo blockade of Type I IFN signaling reduced the recruitment of neutrophils to the lung tissue, mediated the activation of macrophages leading to an increased pro-inflammatory profile and regulated the inflammatory cytokine production. However, no impact was observed on T cell activation and recruitment in the early acute phase of infection. Additionally, blocking of type I IFN signaling reduced bacterial burden in the infected mice as compared to untreated infected mice.

**Conclusions:**

Altogether, our results reveal that Type I IFN mediates a balance between *M. bovis*-mediated inflammatory reaction and host defense mechanism. Thus, modulating Type I IFN signaling could be exploited as a therapeutic strategy against a large repertoire of inflammatory disorders including tuberculosis.

## Background

Tuberculosis is one of the major public health problems and it is caused by *Mycobacterium tuberculosis* (*M. tuberculosis*). According to WHO survey, about 1.3 million deaths and 10 million new cases (out of 1.3 million deaths, 0.3 million were HIV-positive) were reported in 2017 (World Health Organization, 2018) [1]. *Mycobacterium bovis* (*M. bovis*), a member of *Mycobacterium tuberculosis complex* (*MTC*), is the central causative agent of bovine tuberculosis; however, it also infects a wide range of hosts including human beings. Clinically, human tuberculosis caused by *M. tuberculosis* or *M. bovis* is undistinguishable [2].. It has been reported that number of mechanisms are involved in the development of host-immune response against mycobacterial infections [3–6], however, further understanding of the host-pathogen interaction and the intracellular survival of *M. bovis* is necessary for controlling the infection.

The family of Type I IFN comprises of a dozen IFN-α subtypes, IFN-β, as well as IFN-ε, IFN-κ, and IFN-δ which are secreted upon attachment of infectious agents to the pattern-recognition receptors (PPRs) of host cells [7]. All Type I IFN similarly bind to a heterodimeric receptor such as IFNAR1 and IFNAR2; however, the signaling of subtypes of Type I IFN via these receptors results in different functions. In addition, it has been determined that the activity of IFNs depends upon the specificity for the attachment and the potential to induce changes in the receptors [7, 8]. Increasing evidences suggest that IFN-β has a high binding capability to the specified receptors of IFNs for signal generation and the induction of downstream gene expression [9].

Early studies have investigated that Type I IFN contribute to the progression of clinical tuberculosis [10–13]. However, further study is prerequisite to investigate the pathway of Type I IFN signaling that could modulate the host-pathogen interaction during tuberculosis. Therefore, we hypothesized that Type I IFNs may be a modulator of the host immune response against *M. bovis* infection. We found that the expression of Type I IFN and interferon stimulated genes (ISGs) was upregulated in mouse during *M. bovis* infection. Previous studies have demonstrated that high pathogenic strains of *M. tuberculosis* induced an increase production of Type I IFN and subsequently inhibit the expression of pro-inflammatory cytokines leading to abrogation of Th1 responses [14–17]. Reports also suggest that Type I IFN contributes in the recruitment of various myeloid cells which result in the severity of inflammation-dependent lung injury and also promote the dissemination of infection [18, 19]. In addition, drugs that limit Type I IFN induction have been developed to improve the efficacy of tuberculosis treatment [20]. In the current study, we hypothesized that signaling of Type I IFN is crucial in host immune response against *M. bovis* infection. We observed that blockade of Type I IFN signaling reduces the recruitment of inflammatory myeloid cells to the lung tissue, which inhibit the activation of macrophages and result in a pro-inflammatory phenotype thus promoting anti-mycobacterial Th1 response in the early acute phase of infection. Altogether, our findings suggest that therapeutic agents that limit Type I IFN induction could be a better strategy for the control of *M. bovis* infection.

## Methods

### Preparation of cell culture

In the current study, we used murine macrophages (BMDMs and J774a.1 cells). Bone marrow derived macrophages (BMDMs) were obtained from femur and tibia of C57BL/6 mice as described previously [21]. In brief, cells were obtained from femur and tibia of 6 to 8 weeks old mice and cultured in RPMI1640 (Hyclone) medium supplemented with 10% FBS in the presence of 10 ng/ml M-CSF (Pepro Tech) and 1% streptomycin and penicillin (Gibco) in CO_2_ incubator at 37 °C for 7 days. Fresh cell culture medium was added at day 4 of incubation and removed the dead or non-adherent cells. At day 8, the adherent BMDM cells were collected and transferred to 12-well cell culture plates for further experiments. J774a.1 cells were collected from cold storage -80 °C (previously obtained from the Cell Culture Center, Xiehe Medical University (Beijing, China)) and cultured for 2 to 3 days in cell culture flasks in DMEM (Hyclone, Logan, UT, USA) medium in the presence of 10–15% FBS (Gibco, Grand Island, NY, USA), and 1% streptomycin and penicillin (Gibco) at 37 °C in CO^2^ (5%) incubator [22]. After 2 to 3 days, J774a.1 cells were transferred into 12-well culture plates for further experiments.

### Preparation of bacterial culture

In our current experiment, we used virulent strain of *M. bovis*. *M. bovis* Beijing strain C68004 was obtained from China Institute of Veterinary Drug Control (CVCC, China). A stock culture of *M. bovis* was maintained in Middlebrook 7H9 medium (Difco) enriched with 10% albumin-dextrose-catalase (ADC), 2 mg/L sodium pyruvate and 0.05% Tween-80 at 37 °C under biosafety conditions level 3 (BSL3). *M. bovis* was cultured for 2–3 weeks at 37 °C to a concentration of 10^7–8^/ml before used for cells or animal infection.

### Mice model of *M. bovis* infection

C57BL/6 female mice (6–8 weeks of age) were obtained from Vital River Laboratories (Beijing, China). All mice were maintained in a strict biosafety measures in BSL-III laboratory of China Agricultural University, under the protocols of the Laboratory Animal Ethical Committee of China Agricultural University (Protocol 20,110,611–01). Mice were housed in groups as many as 5 mice per cage with free access to feed and water under sterilized condition. Mice were infected with *M. bovis* at 100 CFU (Colony-forming units) via intranasal (i.n) route. The suspension of *M. bovis* bacilli was prepared in sterilized PBS, the negative controlled animals were treated to PBS. Mice were properly anaesthetized by injecting PBS diluted Zoletil 50 (50 mg/kg; Virbac, France) via intraperitoneal route [23] before infection with *M. bovis*. One day post infection (p.i), about five mice were selected randomly and sacrificed by cervical dislocation, under ethics requirements [24], as to calculate viable bacilli in the lung tissues. Next, 6 mice were randomly selected from each experimental group at 21 and 84 day post infection. Blood samples were collected from orbital route [25] of mice after proper anaesthetizing by intraperitoneal injection of PBS diluted Zoletil 50 (50 mg/kg; Virbac, France). Serum samples were obtained from blood by low-speed centrifugation and tissues (lung, spleen) were collected aseptically from all experimental animals and stored at − 80 °C till further experiments.

### In vivo IFNAR1 blocking antibody treatments

In each group, 12 mice C57BL/6 mice were treated twice with 500 μg of anti-IFNAR1 antibody (clone MAR1-5A3; Biolegend San Diego, CA, USA) or mouse IgG1 (500 μg) isotype control (clone MOPC21; Bio X Cell) one day prior to *M. bovis* infection and at 6 weeks post infection through intraperitoneal (i.p) injection. For the enumeration of total viable bacilli, lung and spleen tissues were lysed with small ceramic beads in phosphate buffered saline (PBS), in a tissue homogenizer apparatus (WKT technology) in accordance with the guidelines of manufacturer. An appropriate tenfold serial dilution was prepared in PBS. The dilutions were separately plated in triplicates on Middlebrook 7H11 agar supplemented with ampicillin (10 μg/ml) and sodium pyrovate (2-4 mg/liter). After 2–3 weeks of incubation at 37 °C, *M. bovis* colonies were counted [21].

### IFNAR1 blocking antibody treatment in vitro

To investigate the inhibitory effect of Type I interferon signaling in vitro, BMDMs were treated with 1 μg/ml of IFNAR1 blocking antibody (Biolegend) for 2 h. Then the cells were washed three times with warm PBS and infected with *M. bovis* at multiplicity of infection (MOI) 10 and incubated at 37 °C for 24 h.

### IFN-β production assay

The level of IFN-β in the serum samples was determined by using ELISA assay as previously described (Cusabio, Wuhan, Hubei, China) according to the manufacturer’s protocols [21]. Briefly, 100 μl of standards and samples were added in respective wells of 96 wells antibody coated plate. After incubation and washing steps, conjugated secondary antibodies were added for 1 h followed by same washing steps. Then substrate solution was added in each well followed by addition of stop solution. A standard curve was obtained by using 2-fold dilutions of the standard for each independent experiment. The concentration of cytokines was calculated using a standard curve.

### Multiplex cytokine assays

The lung tissues were homogenized in PBS 0.05% v/v Tween 20 with a supplementation of protease inhibitor cocktail (Roche). The detection of multiple cytokines was carried out by using multiplex bead-based immunoassay kits (Millipore) according to the manufacturer’s protocol. MAGPIX® instrument (Luminex, USA) was used to perform multiplex bead-based immunoassay for the detection of multiple cytokines.

### Quantitative RT-PCR assay

The cDNA synthesis from total RNA (50 ng) was performed by using the Revert Aid first-strand cDNA synthesis Kit (Thermo Fisher Scientific, MA, USA) according to the manufacturer’s protocol. For the quantitative analysis of mRNA, real time-PCR (qRT-PCR) was performed by using AceQ qPCR SYBR Green Master Mix kit (Vazyme Biotech, Nanjing, China) according to the manufacturer’s instructions. β-actin was used as a house keeping gene for data analysis. The sequences of primers used in the current study are mentioned in Table 1. Amplifications were performed with the 700 Fast Real-Time PCR Systems (ViiA7 Real-time PCR, ABI). Thermal cycling conditions were 95 °C for 5 min then 40 cycles at 95 °C for 10 s and 60 °C for 30 s. All fold changes were analyzed by using the ΔΔCt method [22]. Samples were measured in triplicate from three independent experiments.

**Table 1:** Primers used for Quantitative Real-Time PCR

Gene name	Forward primer (5′-3′)	Reverse primer (5′-3′)
*Ifnb1*	AAGAGTTACACTGCCTTTGCCAT	CACTGTCTGCTGGTGGAGTTCATC
*Ifnar1*	CTCCATCTCCTGCTTAGTGT	ATTAGTTACTGTGGGTACTTGTG
*Irf7*	ACTTCAGCACTTTCTTCCGAGAAC	AGGTAGATGGTGTAGTGTGGTGAC
*Oas2*	AAAGTCCTGAAGACCGTCAAGGG	ACAACAATGTCAGCATCTGATCCC
*Igs15*	AGCAATGGCCTGGGACCTAAAG	AGTCACGGACACCAGGAAATCG
*Ifng*	AGCAACAACATAAGCGTCAT	CCTCAAACTTGGCAATACTC
*Tnf*	CAAAATTCGAGTGACAAGCCTGT	CCACTTGGTGGTTTGCTACGA
*Nos2*	TCCTCACGCTTGGGTCTTGTTC	TCCAACGTTCTCCGTTCTCTTGC
*Mrc1*	AATGCCAAAAATTATTGATCGTG	ACGGTGACCACTCCTGCTG
*Ym1*	TACCCTATGCCTATCAGGGTAAT	CCTTGAGCCACTGAGCCTTC
*Arg1*	AATGAAGAGCTGGCCTGGTGTGGT	ATGCTTCCAACTGCCAGACTGTG
*T-bet*	CCCCTGTCCAGTCAGTAACTT	CTTCTCTGTTTGGCTGGCT
*Gata-3*	AAGAAAGGCATGAAGGACGC	GTGTGCCCATTTGGACATCA
*Foxp3*	CTTCACCAAGGTGAGCGAGT	CTTCTGTCTGGAGTGGCTGG
*β-actin*	CCTTCTGACCCATTCCCACC	GCTTCTTTGCAGCTCCTTCG

### Histopathology

For histopathology analysis, the left lung lobe was selected from mice of all experimental groups. 10% formalin buffer was used as a tissue fixative solution and then the lung tissues were sectioned at 5 μm of thickness, followed by H&E or Ziehl-Neelsen staining methods. The inflammatory changes in the lung tissue was measured by observing the H&E stained lung sections under light microscopy at low (× 10) and high (× 40) magnifications. The superior lobes of the left lung were stained with H&E to assess the severity of inflammation; minimum 10 microscopic fields were selected for each section. The level of inflammation in the lung sections was quantified by measuring the area of lesion out of the total area of the section by using ImageJ software (National Institutes of Health, USA).

### Immunohistochemistry

The lung tissues that were already fixed in formalin buffer (10%) were paraffinized and sectioned for immunohistochemical analysis. Briefly, after deparaffinization and antigen retrieval, the lung sections were blocked with BSA for 15 min at 37 °C [26]. After blocking, the sections were incubated at 4 °C with anti-mouse IFNAR1 (Biolegend) or anti-mouse IFN-β (Santa Cruz Biotechnology, CA, USA) overnight followed by HRP-labeled secondary rabbit anti-mouse IgG antibodies (Proteintech, Wuhan, China). The enzymatic activity was revealed by using 3, 3′-Diaminobenzidine (DAB). Digital images were collected on Olympus microscope fitted with DS-Ri2 camera. To quantify the intensity of IFNAR1 and IFN-β, 3 lung sections from independent animals of each group were visualized under low and high power of magnifications. The stained area compared with the total tissue area was determined by using Image-J software.

### Lung cell isolation

Right lung lobes were washed with PBS before excision, and minced on the ice. The minced lung tissues were incubated with DMEM containing 50 μl Collagenase 1 (1 mg/m1) (Solarbio, Beijing, China) and 50 μl DNase 1 (150 U/m1) (Roche Biochemicals) for 1 h at 37 °C and shacked once every 15 min. After that tissue homogenates were passed through a cell strainer of 70 μm pore size (BD Falcon, NY, USA) to make single cell suspension. Lung cells were centrifuged in complete DMEM for 5 min at 1000 rpm for obtaining leukocytes from cell suspension. After centrifugation, the supernatants were removed and the cell pellets were resuspended, and erythrocytes were lysed with Red Blood Cell Lysis Buffer (MultiSciences, Hangzhou, China) by keeping at room temperature for 5 min. About 10 ml of DMEM containing 10% FBS was added to stop the lysis reaction, and after centrifugation the supernatant was removed. After that the cells were resuspended in PBS and the number of cells was counted [26].

### Flow cytometry

For differential quantification of innate immune cells, isolated leukocytes for both lung and spleen tissue were stained with antibodies against Ly6G PerCP-Cyanine5.5 (eBioscience, California, USA), CD11b-APC, CD11c-FITC, Ly6C-PE, CD11c-PE, MHC-II-FITC, CD40-FITC, CD80-FITC, CD86-FITC CD4-FITC and CD8-APC (all from eBioscience). For leukocytes analysis, single-cell suspensions from lungs were stimulated with 10 μg/ml of EAST-6 in the absence or presence of 4 μg/ml brefeldin A (Sigma, St. Louis, USA) for 6 h at 37 °C [26]. CD206-PE (eBioscience) antibody was used for the detection of intracellular markers of lymphocyte subsets.

### Statistical analysis

The data was analyzed by using GraphPad Prism 5 software USA. Student t test was applied for comparing between two groups. One way or two way ANOVA was applied for the comparison of multiple groups.

## Results

### The induction of type I interferon during *M. bovis* infection

**Fig. 1:**
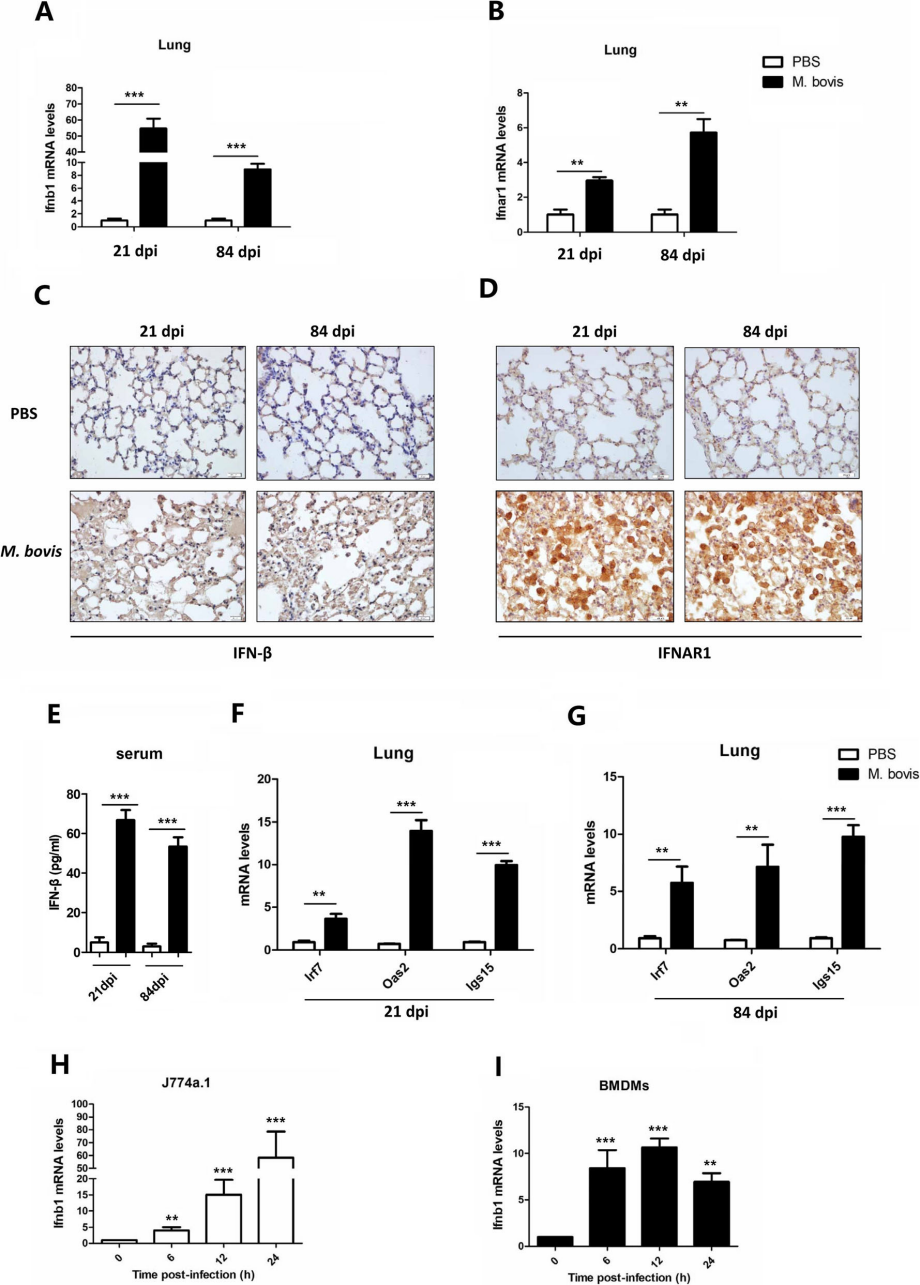
*M. bovis* induces type I interferon in C57BL/6 mice. (**b**-**e**)Wild type female C57BL/6 mice were challenged with M. bovis (100 CFU) or PBS by intranasal (i.n) route. Mice (n = 3) from each group were analyzed for gene and protein expression at 21 and 84 days post-infection. (**a**) Ifnb1 and (**b**) Ifnar1 mRNA expression in the lung tissues were checked by qRT-PCR. Gene expression values were normalized to the housekeeping gene β-actin. (**c**) IFN-β and (**d**) IFNAR1 expression in the lungs were detected by IHC method; scale bars, 20 μm. Original magnification, 100×. (**e**) The level of IFN-β in the blood serum was determined by ELISA. mRNA isolated from lung tissues was probed for the expression of indicated genes by qRT-PCR at (**f**) 21 dpi and (**g**) 84 dpi. (**h**) J774A.1 and (**i**) BMDM cells were infected with M. bovis at a multiplicity of infection (MOI) of 10 for the indicated time, mRNA was isolated and probed for the expression of Ifnb1 gene by qRT-PCR. Data were normalized to housekeeping gene β-actin. Data was obtained from three independent experiments for in vitro study. Data is presented as mean±SD (* *P < 0.05;* ** *P < 0.01;* *** *P < 0.001*)

In order to identify Type I interferon signaling that is critical for the pathogenesis of *M. bovis*, we infected C57BL/6 mice with virulent *M. bovis* by intranasal route (i.n). After 21 and 84 days of infection, mice were sacrificed, and lungs, spleen and serum samples were collected aseptically for analysis of IFN-β and IFNAR1. We observed a significant increased level of mRNA expression of *Ifnb1* and *Ifnar1* in the lung (Fig. 1a and b) and spleen tissue (Additional file 1: Figure S1A and S1B) at both 21 and 84 days after infection in comparison with uninfected PBS control mice. Similarly, the protein level of IFN-β and IFNAR1 was also significantly high in the lungs and spleen tissue of *M. bovis* infected mice than PBS controls at both 21 and 84 days post-infection (Fig. 1c and d, Additional file 1: Figure S1C and D). In addition, we found an increased level of IFN-β in the serum of *M. bovis* infected mice than PBS control mice (Fig. 1e), as well as IFN-responsive genes such as *Irf7*, *Oas2* and *Isg15* at the indicated time points (Fig. 1f and g, Additional file 1: Figure S1E and S1F).

Next, we infected J774A.1 and BMDM cells with *M. bovis* to determine the expression of *Ifnb1*. The quantitative real time PCR results revealed a significant increase in the expression of *Ifnb1* at 6, 12, and 24 h post-infection (Fig. 1h and i). Together, these results suggest that *M. bovis* induces the production of Type I IFN during infection.

### Type I IFN signaling contributes to the pathogenesis of *M. bovis*

**Fig. 2:**
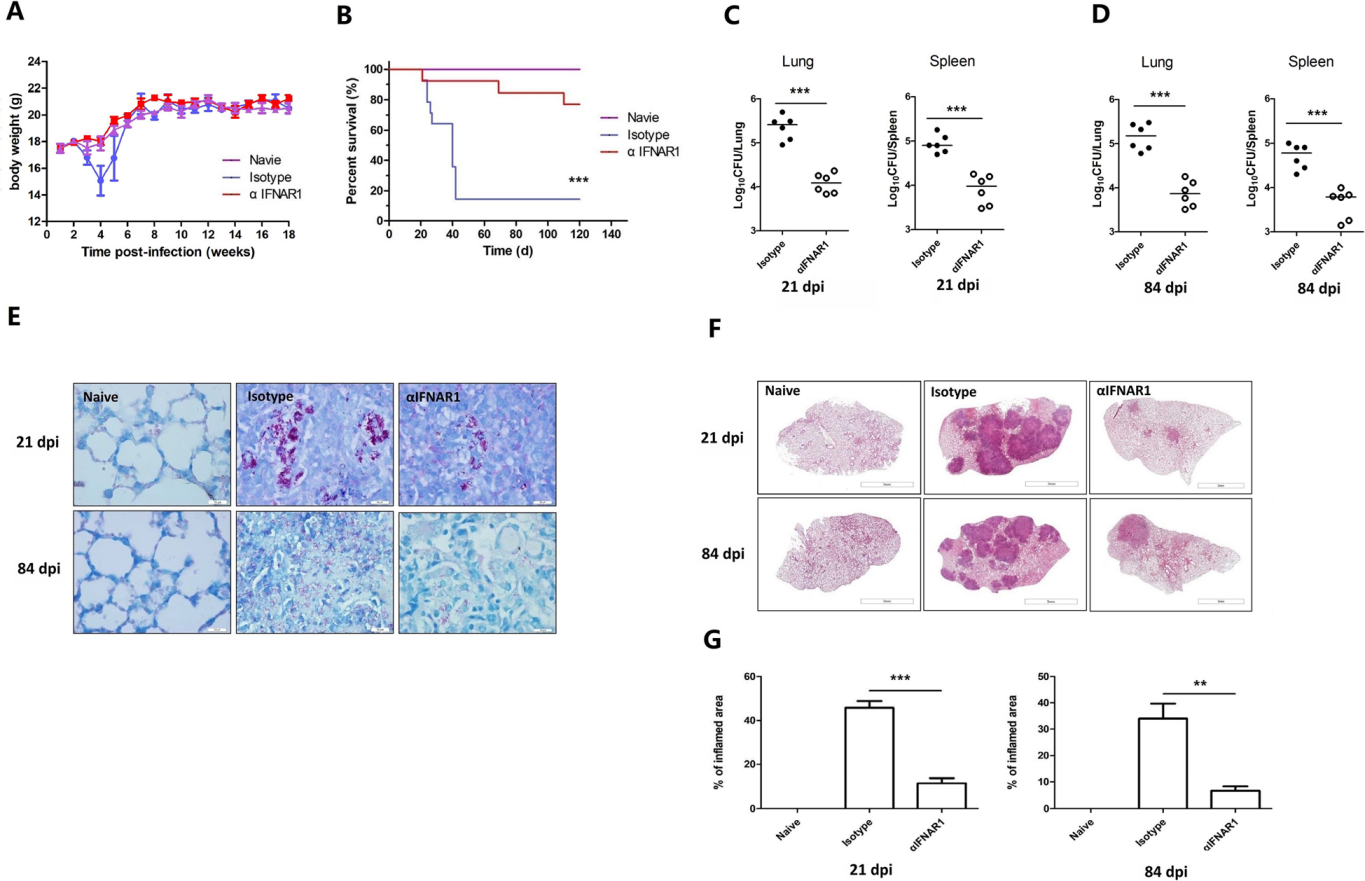
IFNAR1 blockade reduces the severity of *M. bovis* infection in mice. C57BL/6 mice were treated with αIFNAR1 antibody or isotype antibody followed by infection with 100 CFUs of *M. bovis* via i.n route. **a** Body weights of mice with IFNAR1 antibody or mouse IgG1 isotype antibody (*n*  =  6 mice). Data presented from two experiments. **b** Survival curve (obtained from 2 independent trials, *n*  =  18 for mice with mouse IgG1 isotype control antibody, *n*  =  18 for mice with IFNAR1 antibody); *P  < 0.001* compared to mice with IgG1 isotype antibody, analyzed by Log-rank (Mantel-Cox) test. **c** and **d** Bacterial load from lung and spleen at 21 and 84 days pi; data shown are representative of two independent trials, *n*  =  6 mice per group. **e** Acid-fast staining of lung sections of *M. bovis* infected mice (scale bar: 10 μm). **f** and **g** H&E staining of paraffin-embedded tissue collected at 21 and 84 days p.i for histopathology analysis (scale bar, 2 mm; *n* = 5). Data is presented as mean ± SD from two independent experiments

We further investigated the role of Type I IFN in the pathogenesis of *M. bovis*, and found that IFNAR1 blockade diminished IFN-responsive *Irf7, Oas2 and Isg15* genes expression in the lung and spleen tissues (Additional file 2: Figure S2A and S2B), indicating its ability to inhibit Type I IFN signaling*.* IFNAR1 blockade also decreased IFN-β production in blood serum of *M. bovis* infected mice (Additional file 2: Figure S2C). Next, we examined the susceptibility of C57BL/6 mice to pulmonary infection by *M. bovis* after treatment with IFNAR1-blocking antibody (αIFNAR1 mice) or mouse IgG1 isotype control antibody (Isotype mice). Initially, we evaluated the survival rate of αIFNAR1 and Isotype mice after intraperitoneal infection with a low dose (100 CFUs) of *M. bovis.* We found that αIFNAR1 treated mice were less sensitive to infection, with a significant decrease in mortality rate and increased survival time after infection. In addition, αIFNAR1 mice survived acute (day 21 post-infection) and chronic infection (day 84 post-infection), while Isotype treated mice rapidly lost their body weight and half of the mice were died within 40 days post-infection (Fig. 2a and b). Next, we measured total viable *M. bovis* bacilli in the lung and spleen of infected mice at various time points post infection. We found significantly reduced viable bacilli in the lung and spleen tissues of αIFNAR1 treated mice as compared to Isotype treated group at both 21 and 84 days post infection (Fig. 2c and d). Similarly, reduced number of Acid-fast *M. bovis* bacilli were observed in lung sections of αIFNAR1 treated mice as compared with Isotype treated mice (Fig. 2e). Histological examinations of lungs sections revealed that granulomatous inflammatory lesions were significantly sever in the Isotype treated mice than αIFNAR1 mice (Fig. 2f and g). Collectively, these findings demonstrate that Type I IFN signaling induced by *M. bovis* significantly promotes bacterial proliferation and contributes towards the pathogenesis of *M. bovis* infection in mice.

### Type I IFN signaling mediates lung inflammatory cytokines that correlate with disease progression

**Fig. 3:**
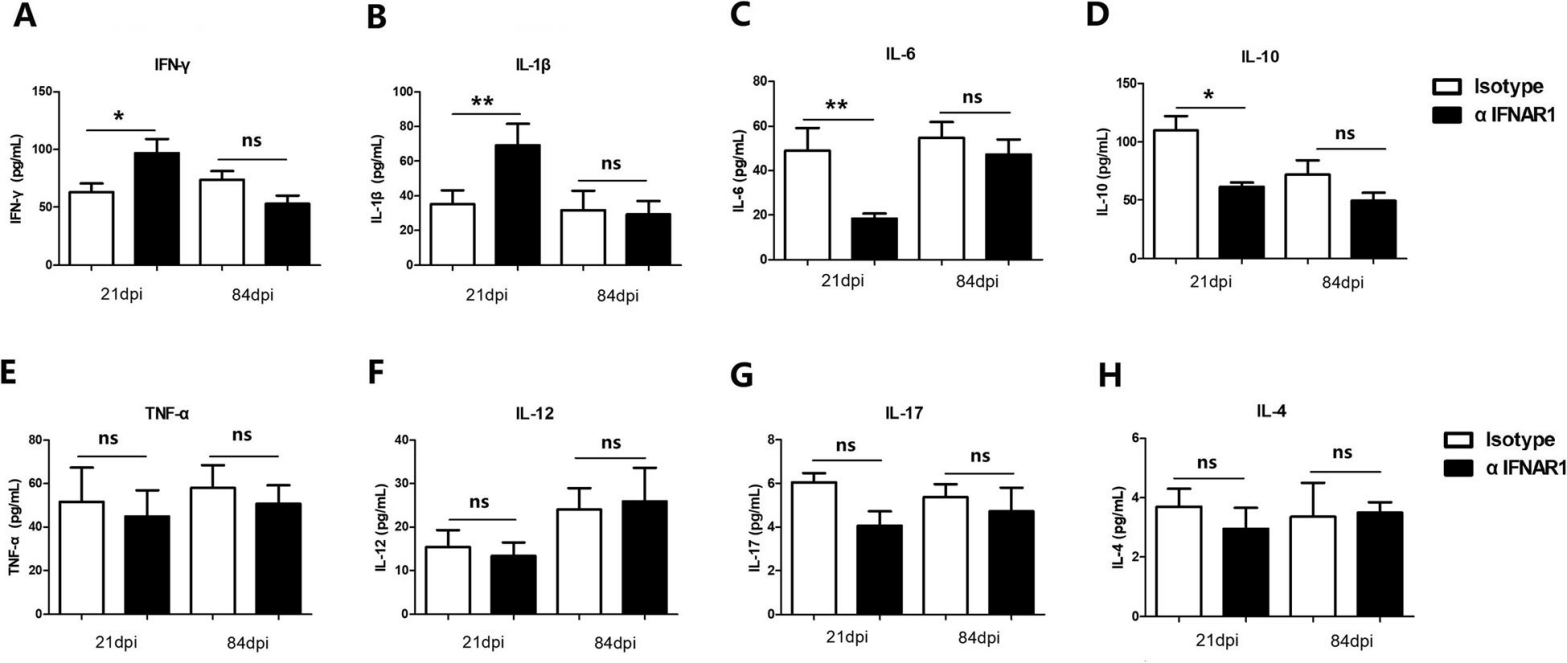
The effect of IFNAR1 blockade on cytokine production in *M. bvois* infected mice. (A-H) lung homogenates from mice treated with αIFNAR1 or isotype antibody followed by *M. bovis* infection was analyzed for cytokines production at 21 and 84 days p.i (*n* = 6); (**a**) IFN-γ, (**b**) IL-1β, (**c**) IL-6, (**d**) IL-10, (**e**) TNF-α, (**f**) IL-12, (**g**) IL-17, (**h**) IL-4. Data shown is presented as mean ± SD from two independent experiments. (* *P < 0.05;* ** *P < 0.01*)

Next, we evaluated the effect of Type I IFN signaling on the regulation of various cytokines. We quantified the levels of multiple cytokines in lung tissue samples by using multiplex bead-based immunoassay kits. We found significantly high levels of IFN-γ and IL-1β in αIFNAR1 treated mice while low levels were observed in Isotype mice at 21 days post-infection (Fig. 3a, b). In contrast, the level of IL-10 and IL-6 in αIFNAR1 treated mice was decreased as compared to Isotype mice at day 21 post-infection (Fig. 3c, d). Interestingly, we also found no significant difference in the production of IL-12, TNF-α, IL-17 and IL-4 at day 21 post infection between αIFNAR1and Isotype treated mice. (Fig. 3e-h). Similarly, at day 84 post-infection, no clear difference was observed in the concentration of IFN-γ, IL-1β, TNF-α, IL-12, IL-6, IL-10 and IL-4 in the both groups of mice (Fig. 3a-h). These results indicate that Type I IFN signaling contributes towards inflammatory reactions during early *M. bovis* infection.

### Type I IFN signaling regulates the accumulation and activation of myeloid cells in the lung during *M. bovis* infection

**Fig. 4:**
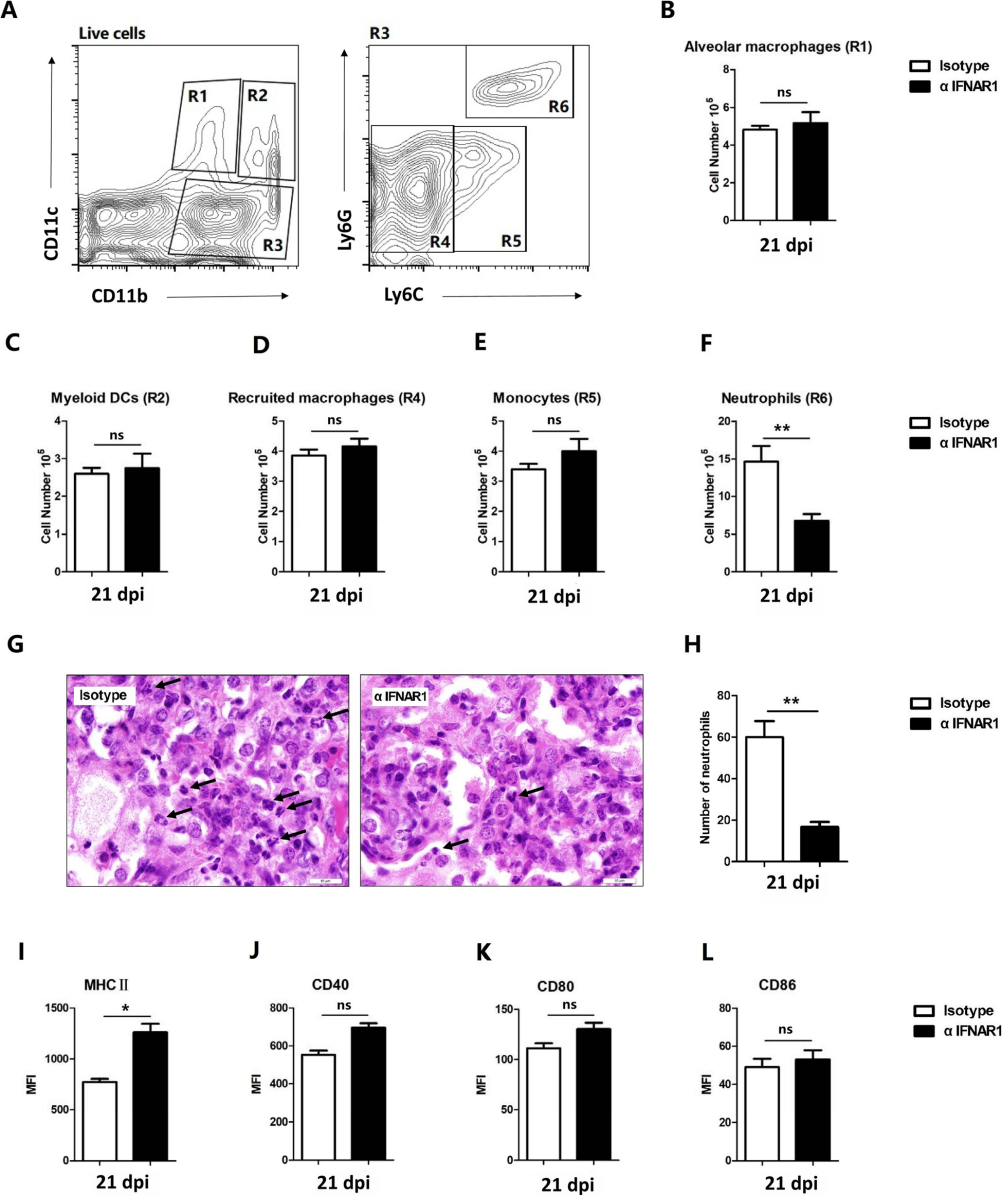
Type I IFNs regulates lung myeloid cell recruitment during *M. bovis* infection. Mice treated with αIFNAR1 antibody or isotype antibody followed by infection with *M. bovis*. Myeloid cell populations in infected lung tissues were analyzed by flow cytometry at 21 days p.i. **a** Lung cells were gated on single live cells using forward and side scatter parameters. Gating strategy for quantification of myeloid cell populations is shown. R1 (CD11b^lo^ CD11c^+^): alveolar macrophages; R2 (CD11b^hi^ CD11c^+^): myeloid dendritic cells (DCs); gating on R3 (CD11b^hi^ CD11c^neg^), R4 (CD11b^hi^ CD11c^neg^ Ly6C^lo/neg^): recruited macrophages; R5 (CD11b^hi^ CD11c^neg^ Ly6C^int/hi^ Ly6G^neg^): monocytes; and R6 (CD11b^hi^ CD11c^neg^ Ly6C^int^ Ly6G^hi^): neutrophils. **b**-**f** Quantification of myeloid cells phenotypes. Each bar represents mean ± SD (n = 5). Data are a pool of two independent experiments. **g** H&E staining of lung sections were performed to measure neutrophils infiltration (scale bar: 10 μm). Arrows: neutrophils. **h** The number of neutrophils infiltrating the lungs of αIFNAR1 mice was evaluated at 21 days p.i. (**I**) Expression of MHC class II on myeloid DCs in the lungs of mice at 21 days p.i. Data are expressed as MFI ± SE (*n* = 3). **j**-**l** Expression of costimulatory molecules, (**j**) CD40, (**k**) CD80, and (**l**) CD86 on myeloid DCs in the lungs of mice at 21 days p.i; Data are expressed as MFI ± SE (*n* = 3). (* *P < 0.05;* ** *P < 0.01*)

Our approach, based on Type I IFN receptor blocking antibodies, allowed us to examine how Type I IFN signaling affects lung myeloid cell populations during *M. bovis* infection. As shown by flow cytometry (Fig. 4), we observed reduced lung pathology associated with reduction in the recruitment of neutrophils into the lung tissue of αIFNAR1 mice, though no significant difference was found in the population of alveolar macrophages, myeloid DCs, recruited macrophages and monocytes between αIFNAR1 and Isotype mice (Fig. 4a-f). Consistent with flow cytometry assay, histological assay revealed a noticeable reduction in the population of neutrophils in the lung sections of αIFNAR1 mice at day 21 post-infection (Fig. 4g and h). These findings suggest that Type I IFN produced locally in lungs plays a significantly role in the migration of neutrophils to the lungs in the early phase of *M. bovis* infection. The blockade of αIFNAR1 rescue mice from adverse inflammatory reactions by preventing the excessive migration of neutrophils to the lung tissue.

Next, we sought to determine the expression of MHC class II and co-stimulatory molecules on myeloid cells. We observed that αIFNAR1 mice showed a significantly high level of MHC class II expression on myeloid DCs compared with Isotype mice at 21 days post-infection (Fig. 4i). On contrary, IFNAR1 blockade had no noticeable effect on the expression levels of CD40, CD80 and CD86 (Fig. 4j-l). Our observations reveal that IFNAR1 blockade decreased the number of recruited neutrophils and lead to a weaker activation of myeloid DCs in the lungs during early *M. bovis* infection.

### Type I IFN signaling mediates macrophage polarization toward an anti-inflammatory profile during *M. bovis* infection

**Fig. 5:**
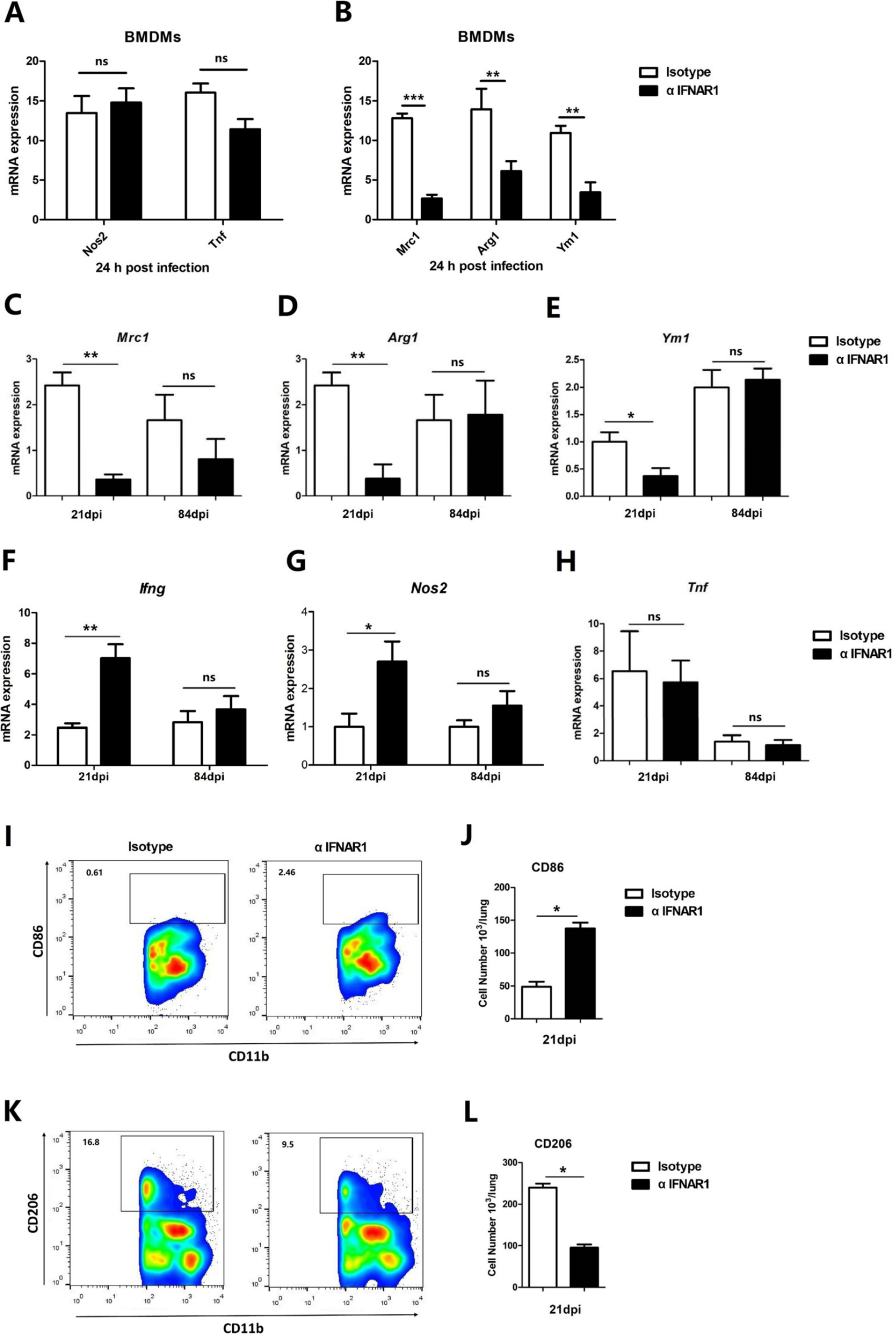
Type I IFNs drive macrophage polarization toward an anti-inflammatory profile in *M. bovis*-infected lungs. **a**-**b** BMDMs were infected with *M. bovis* at a multiplicity of infection (MOI) of 10 in the presence or absence of the αIFNAR1 antibody. Relative expression of macrophage M2 (*Arg1*, *Ym1*, and *Mrc1*) and M1 (*Nos2* and *Tnf*) polarization gene markers were quantified by qRT-PCR at 24 hpi. Data were normalized to housekeeping gene β-actin. Mice were infected with *M. bovis* as mentioned above (Fig. 4). **c**-**e**) qRT-PCR was performed for quantification of M2 (*Arg1*, *Ym1*, and *Mrc1*) and (**f**-**h**) M1 (*Ifng*, *Nos2* and *Tnf*) polarization markers in the lungs of infected mice. The effect of αIFNAR1 antibody treatment on polarization of macrophages in the lung tissues of mice infected with *M. bovis* at 21 days p.i. Representative flow cytometric plots of CD86-expressing (M1) (**i**-**j**) and CD206-expressing (M2) macrophages and relative cell numbers (**k**-**l**). Each bar represents mean ± SD (*n* = 5). Data are a pool of two independent experiments

In the next experiment, we assessed whether the signaling mechanism of Type I IFN could affect macrophage polarization during *M. bovis* infection. BMDMs treated with αIFNAR1 antibody exhibited no difference in the expression of the M1 markers such as *Nos2* (nitric oxide synthase 2) and *Tnf* as compared to control group at 24 h post-infection (Fig. 5A). In contrast, these macrophages displayed a decreased expression of the M2 markers such as *Arg1*, *Ym1*, and *Mrc1* (mannose receptor C-type 1) at 24 h post-infection (Fig. 5b). These findings show that Type I IFN induced by *M. bovis*-activated macrophages can directly shift the profile of macrophages into anti-inflammatory phenotype.

The increased level of Type I IFN leads to the attenuation of macrophage phenotypes in the lungs of *M. bovis*-infected mice. Early study reported that increased production of Type I IFN is associated with clinical tuberculosis [10], therefore, we determined the effect of blocking of Type I IFN signaling in the *M. bovis*-infected mice. Gene analysis in the lung tissues of αIFNAR1 mice showed a decreased in the expression of the M2 markers such as *Arg1*, *Ym1*, and *Mrc1* (Fig. 5c-e), while high expression level of M1 markers such as *Nos2* and *Ifng* were found compared with Isotype mice at 21 days post-infection (Fig. 5f-h) but no difference was observed at 84 days post infection.

In addition, flow cytometric analysis of the CD11b^+^ F4/80^+^ macrophage population in the lung tissue of αIFNAR1 mice revealed an increased population of CD86^+^ (M1) (Fig. 5i, j), while substantially reduced population of CD206^+^ (M2) macrophages (Fig. 5k, l). These observations are in line with our initial hypothesis that loss of Type I IFN signaling results in macrophage polarization toward a pro-inflammatory profile in infected lung tissues.

### The effect of type I IFNs on T cell recruitment and activation during early *M. bovis* infection

In order to evaluate the effect of Type I IFNs during *M. bovis* infection on priming or trafficking of T lymphocytes, we examined populations of CD4^+^ and CD8^+^ T cells in the lung and spleen tissues of infected αIFNAR1 or Isotype treated mice. Flow cytometry results revealed that at both 21 and 84 days post-infection, no significant difference was observed in CD4^+^ and CD8^+^ T cell populations in the lung and spleen tissues of αIFNAR1 and Isotype treated mice (Additional file 3: Figure S3A, S3B).

## Discussion

The distribution of tuberculosis is worldwide and it remains one of the deadliest infectious diseases of human and animals. Increasing evidence suggests that cytokines are key mediators in host protective response against *M. tuberculosis* infection [27]. It has been reported that IFN-γ [27], TNF-α [28], and IL-12 [29] are critical cytokines in controlling *M. tuberculosis* infection in mouse model of tuberculosis. On the other hand, emerging studies suggests that Type I IFN plays a critical role in chronic tuberculosis. Though several studies have reported that Type I IFN contribute in the activation of host immune cells during in vitro infection of *M. tuberculosis*, a number of other studies have also reported the adverse impact of these cytokines on the recovery of infection in vivo [14–16]. In the current study, we addressed this issue by investigating the effects of the IFNAR1 antibody in the regulation of Type I IFN in *M. bovis* infection both in vivo and in vitro. These observations suggest that the inhibition of IFNAR1 significantly extenuates pulmonary immunopathology, reduced the recruitment of neutrophils to the lung, modulate the polarization of macrophages, and promote anti-mycobacterial Th1 response during *M. bovis* infection. Our observations are in line with early studies showing a key role of host innate immune cells in investigating the outcome of mycobacterial infection in vivo [30, 31].

In the previous studies, IFN-depended gene signatures were observed in the blood of acute phase of tuberculosis, as well as, in the patient of chronic stage of infection from UK, Indonesia and South Africa [10, 11, 32]. In addition, it is reported that blood transcriptional profiles of individuals in the early stage of tuberculosis were dominated by Type I IFN-inducible gene profiles, that were associated with radiographic detectable lung lesions and subsided with proper therapy [10]. In addition, high numbers of IFN-mediated signatures were also found in mice infected with *M. tuberculosis*, as well as, in *M. bovis*-infected cattle [13, 32–34]. Similarly, our results showed that Type I IFN and Type I IFN-mediated genes were significantly increased in the lung and spleen tissues of mice challenged with *M. bovis* as compared to uninfected control mice. Overexpression of Type I IFN in the mouse infected with *M. tuberculosis* has shown the adverse effects of Type I IFN signaling during tuberculosis. It has been studied that direct instillation of purified murine IFN-α/β into the lungs during *M. tuberculosis* infection is highly deleterious to the host [14]. Similarly, increased induction of IFNα/β production during *M. tuberculosis* infection after treatment with TLR3 agonist results into enhanced bacterial burdens and exacerbated pulmonary immunopathology [18, 20]. Likewise, blockade of counter regulators of Type I IFN signaling, MAPK kinase kinase 8 (MAP 3 K8; also known as TPL2), that activates signaling downstream of TLRs, results into over-production of Type I IFN that attenuated the host-immune defense mechanism to reduce the growth and survival of *M. tuberculosis* or *L. monocytogenes* [35, 36]. Conversely, these studies indicated the absence of Type I IFN signaling is associated with a decreased bacterial burden. Similarly, we found that during *M. bovis* infection, lung lesions were overall extenuated by the blockade of Type I IFN signaling and the pathological severity and host survival was correlated with bacterial load.

The signaling cascades mediating the regulation of Type I IFN, associated with the severity of disease, are not fully elucidated. Reports demonstrated that Type I IFN contributes to host-susceptibility to *M. tuberculosis* infection by different mechanisms such as production of IL-10 and counter regulation of IL-1β/PGE2, and IL-12/IFN-γ axis mediates protective immune responses. Furthermore, Type I IFN inhibits host immune defense mechanism by negative regulation of IL-1α and IL-1β in tuberculosis [37–39]. Recently, it has been reported that IL-1β negatively regulates the production of Type I IFN through prostaglandin E2 (PGE2)-mediated mechanism. By promoting the levels of PGE2 during *M. tuberculosis* infection, IL-1β leads to inhibition of Type I IFN production thus contributes to reduce the susceptibility of mice to *M. tuberculosis* infection [20]. In our study, we found that blockade of Type I IFN signaling reduced the production of IL-10 while enhanced the production of IL-1β in lung homogenates during early stage of *M. bovis* infection. Previous studies have reported that IL-12 and TNF-α production was negatively regulated in human monocytes treated with IFN-α or IFN-β [40] and similar findings were observed in murine macrophages [39]. In addition, it is reported that Type I IFN induce the production of the anti-inflammatory cytokine, IL-10 in macrophages [37, 39]. Furthermore, the negative regulation of IL-12 and TNF-α by Type I IFN signaling was blocked in the absence of IL-10 in *M. tuberculosis*-infected macrophages [39]. It is also known that *M. tuberculosis* actively suppresses the production of IL-12 and TNF-α in macrophages, independent of Type I IFN signaling, by the secretion of ESAT-6/CFP-10 [33]. It is broadly accepted that IL-12 and TNF-α both are essential for host resistance against infection but overproduction of these cytokines may contribute towards immunopathology. Our results showed that blocking of IFNR1 did not affect IL-12 and TNF-α production during *M. bovis* infection. In addition, Type I IFNs can also down regulate IFN-γ signaling by inhibiting IFNGR1 suggesting a detrimental role of Type I IFNs in the pathogenesis of *M. tuberculosis* via antagonizing host-protective functions of IFN-γ [39–41]. In our current study, we observed that blockade of Type I IFN signaling promotes the production of IFN-γ in lung homogenates during early stage of *M. bovis* infection. Collectively, these results reveal that Type I IFN may contribute to host-pathogen interaction locally by regulating the induction of host-protective cytokines at the site of infection.

It has been unveiled that Type I IFN increase the susceptibility of alveolar macrophages [19] and recruit immunologically exhausted myeloid cells to the site of infection, which contribute to the spread of infection and pulmonary inflammation [18, 19]. In addition, enhanced IFN-mediated genes in the blood neutrophils and monocytes were detected, but not in CD4^+^ or CD8^+^ T cells, in patients with active phase of tuberculosis [10, 42] suggesting the role of monocytes and neutrophils in the Type I IFN-mediated pathogenesis of tuberculosis. Panteleev and colleagues reported that neutrophils have a positive correlation with the extent of lung injury, bacterial dissemination, and “Timika X-ray score” [43]. These features illustrate that neutrophils are not efficient to inhibit mycobacterial replication in comparison to residing macrophages of lung. It also implicates that the existence of neutrophils in TB associated inflammation promotes the induction of lung pathology rather than protection thus neutrophils may serve as a “Trojan horse” for the dissemination of mycobacteria [44]. In consistent with these results, we found significantly lower number of neutrophils at 21 days after *M. bovis* infection in αIFNAR1 treated mice. These findings suggest that Type I IFN participates in the development of acute inflammation in the lung tissue mediated by neutrophils during *M. bovis* infection in mice.

The polarization of macrophages into M1 class is specifically a pro-inflammatory phenotype with anti-bacterial ability while M2 class of macrophages are specialized in tissue repairing and also resist to helminths infection [45]. It has been reported that B cell-restricted Myd88 deficient mice show an increased M2 macrophages in the lungs tissue during *M. tuberculosis* infection which is associated with increased bacterial load [46]. Furthermore, C-type lectin receptor (DCIR) deficiency results in inhibition of Type I IFN signaling which in turn leads to macrophage activation and polarization into antimicrobial M1 phenotype [47]. In line with these results, we noticed that blockade of Type I IFN signaling modulates macrophage polarization towards a proinflammatory profile. Collectively, these results reveal that innate signaling in the lung mediated by Type I IFN production, leads to polarization of macrophage into M2-like profile in the early stage of *M. bovis* infection.

Previously, IFN-β has been used for the control of multiple sclerosis, possibly via inhibitory effect on the function of T helper 1 (Th1) cells, but the detailed mode of action is not fully elucidated yet [48, 49]. In the case of TB, inhibition of Type I IFN signaling in DCs mediated by STAT1 results into high expression of IL-12 and also stimulate the differentiation of T cells into Th1 cell population [47]. However, Type I IFN signaling also mediates the survival of activated CD4 T cells and its role in the activation of effector CD8 T cells has been investigated in vaccination programs [50]. In addition, recombinant Type I IFN shows direct adjuvant activity in vivo contributing to the both humoral and T cell mediated immune responses [51, 52]. However, the severity of *M. tuberculosis* infection by Poly-ICLC is not owing to the attenuation of effector Th1 cell function [18]. Evidence suggests that mice deficient in IFNAR1 are more resistant to the infection of *M. tuberculosis* than WT mice, while no significant effect on T cells function was observed [19]. Quantitative analysis of antigen-specific Th1 cells parameters revealed that Th1 cells response play a minor role in determining the severity of tuberculosis [43]. As depicted by our current data, *M. bovis* exploits Type I IFN signaling pathway to evade the host immune response but it does not alter the T cells recruitment and activation in the lung and spleen tissues.

## Conclusion

In conclusion, we found that blockade of Type I IFN signaling reduces the recruitment of neutrophils to the lung, modulates the polarization of macrophages toward a pro-inflammatory profile, inhibits the production of IL-10, promotes the production of IFN-γ and IL-1β, and has no effects on T cells function in the early acute phase of the disease. Moreover, Type I IFN signature gene phenotype provides a potential diagnostic tool and may have implications for vaccine development, and can be exploited as a target in the host-directed therapy to combat mycobacterial infection.

## Supplementary information

**Additional file 1: Figure S1.** *M.bovis* induces type I interferon production in C57BL/6 mice. (A-C) Wild type female C57BL/6 mice were challenged by i.n route with 100 CFU of *M. bovis* or PBS. (A) IFN-β and (B) IFNAR1 expression in the spleen tissue were detected by qRT-PCR. (C and D) Representative images of spleen sections for (C) IFN-β and (D) IFNAR1 expression by IHC method; scale bars, 20 μm. (E and F) IFN-responsive genes such as *Irf7*, *Oas2* and *Isg15* were checked by qRT-PCR at (E) 21 and (F) 84 days p.i. Gene expression values were normalized to the housekeeping gene β-actin. Data is presented as mean ± SD (*n* = 3) (* P < 0.05; ** P < 0.01; *** P < 0.001).

**Additional file 2: Figure S2.** IFNAR1 blockade inhibits Type I IFN signaling in vivo. C57BL/6 mice were treated with αIFNAR1 or isotype antibodies one day prior to *M. bovis* infection. (A and B) The relative mRNA expression of *Oas2, Isg15* and *Irf7* were calculated by qRT-PCR in (A) lung and (B) spleen (*n* = 5). (C) The concentration of serum IFN-β was calculated by ELISA at 21 days p.i (n = 5). Data is presented as mean ± SD from two independent experiments.

**Additional file 3: Figure S3.** IFNAR1 blockade do not affect the population of CD4^+^ or CD8^+^ T cells in vivo. C57BL/6 mice were treated with αIFNAR1 or isotype antibodies one day prior to *M. bovis* infection. At 21and 84 days p.i, cell suspensions from lung and spleen were stimulated in vitro with ESAT-6:1–20 (10 μg/ml). (A-B) Representative flow cytometric plots of CD4^+^ or CD8^+^ T cells by ICS applied on total (right panel) lung cells and (left panel) splenocytes of mice at 21 and 84 days p.i (n = 3). Data is presented from two independent experiments.

## Data Availability

All data generated or analyzed during this study are included in the manuscript and the supporting information are available in the supplementary materials.

## Abbreviations

BSL3: Biosafety conditions level 3
CFU: Colony forming units
ELISA: Immunosorbent assay
H&E: Hematoxylin and eosin
IHC: Immunohistochemistry
IL-10: Interleukin-10
*M. bovis*: *Mycobacterium bovis*
*M. tuberculosis*: *Mycobacterium tuberculosis*
PCR: Polymerase chain reaction
TB: Tuberculosis
Type I IFN: Type I interferon
