# Supplementary figures and images for: Inhibition of type I interferon signaling abrogates early Mycobacterium bovis infection
Source: BMC Infect Dis. 2019 Dec 4;19:1031. doi: 10.1186/s12879-019-4654-3 (PMC6894119; doi:10.1186/s12879-019-4654-3)

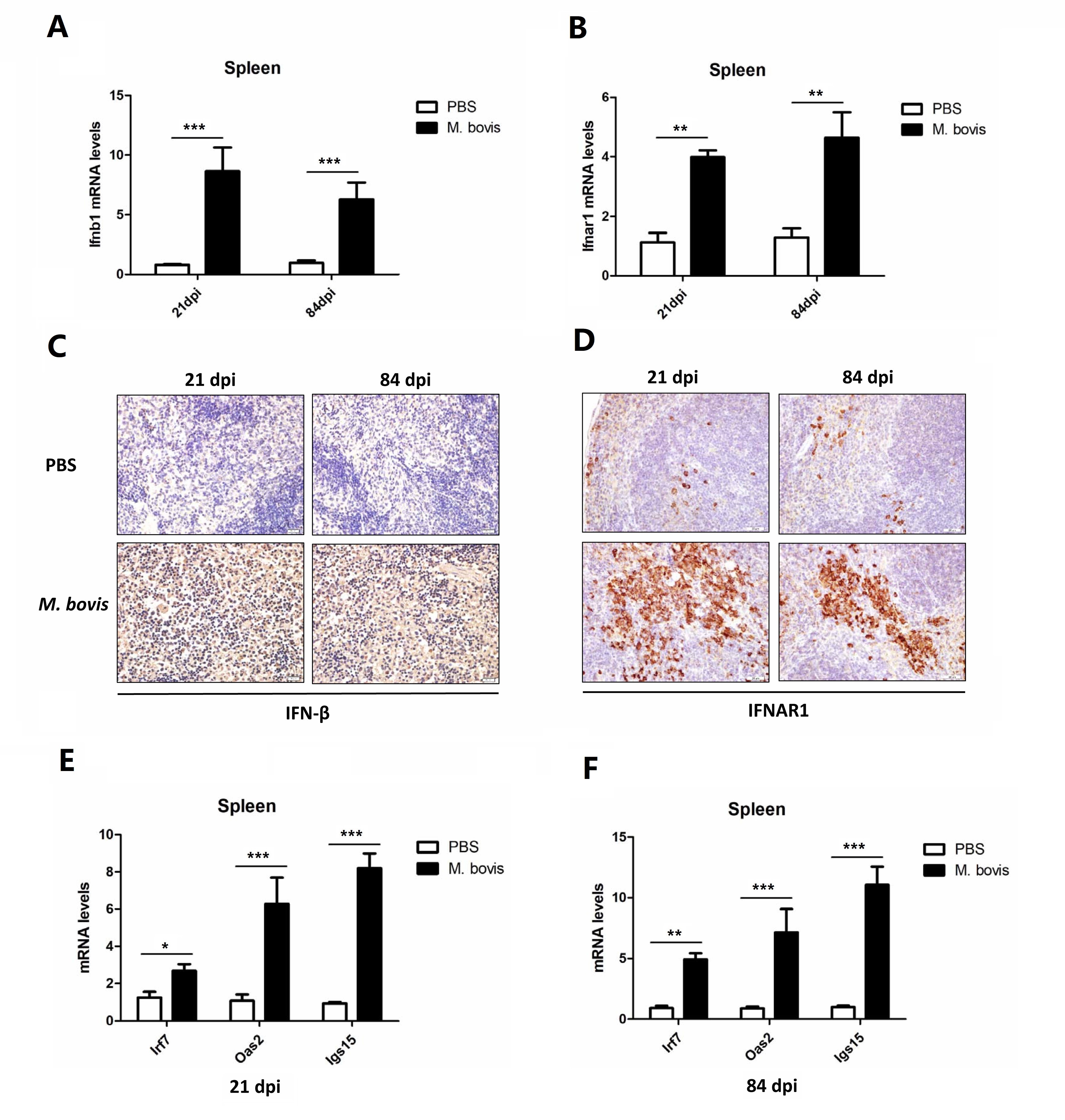

Supplement: Supplementary file 1 — Additional file 1: Figure S1. M.bovis induces type I interferon production in C57BL/6 mice. (A-C) Wild type female C57BL/6 mice were challenged by i.n route with 100 CFU of M. bovis or PBS. (A) IFN-β and (B) IFNAR1 expression in the spleen tissue were detected by qRT-PCR. (C and D) Representative images of spleen sections for (C) IFN-β and (D) IFNAR1 expression by IHC method; scale bars, 20 μm. (E and F) IFN-responsive genes such as Irf7, Oas2 and Isg15 were checked by qRT-PCR at (E) 21 and (F) 84 days p.i. Gene expression values were normalized to the housekeeping gene β-actin. Data is presented as mean ± SD (n = 3) (* P < 0.05; ** P < 0.01; *** P < 0.001). [file 12879_2019_4654_MOESM1_ESM.jpg]

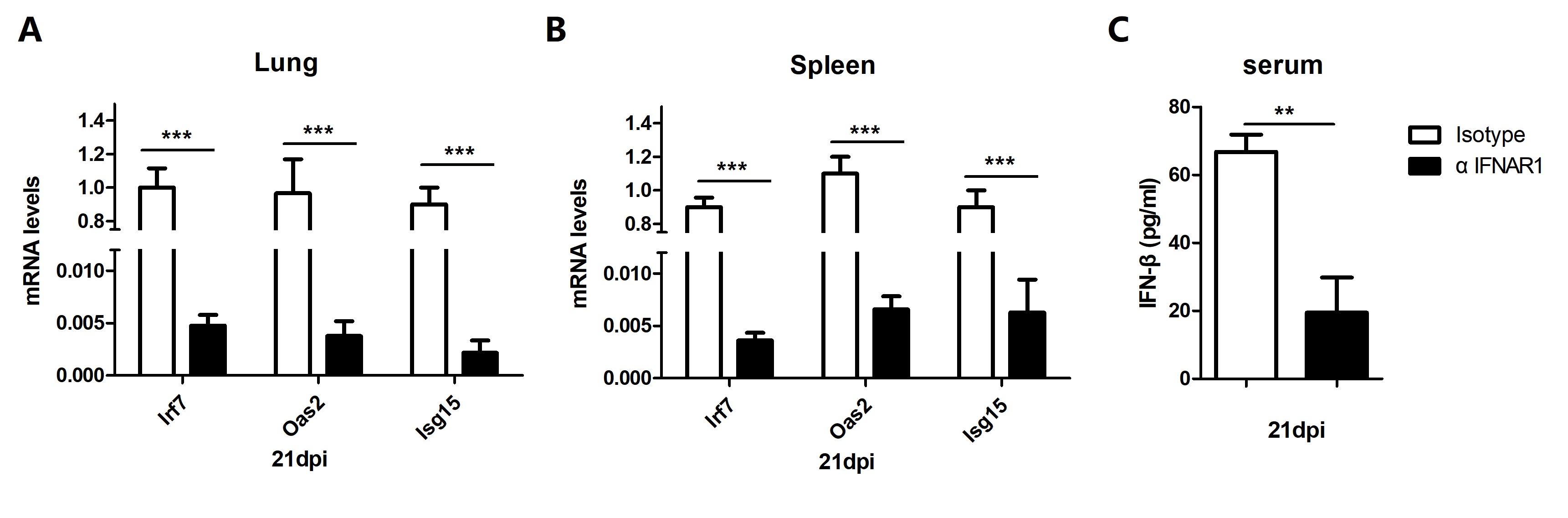

Supplement: Supplementary file 2 — Additional file 2: Figure S2. IFNAR1 blockade inhibits Type I IFN signaling in vivo. C57BL/6 mice were treated with αIFNAR1 or isotype antibodies one day prior to M. bovis infection. (A and B) The relative mRNA expression of Oas2, Isg15 and Irf7 were calculated by qRT-PCR in (A) lung and (B) spleen (n = 5). (C) The concentration of serum IFN-β was calculated by ELISA at 21 days p.i (n = 5). Data is presented as mean ± SD from two independent experiments. [file 12879_2019_4654_MOESM2_ESM.jpg]

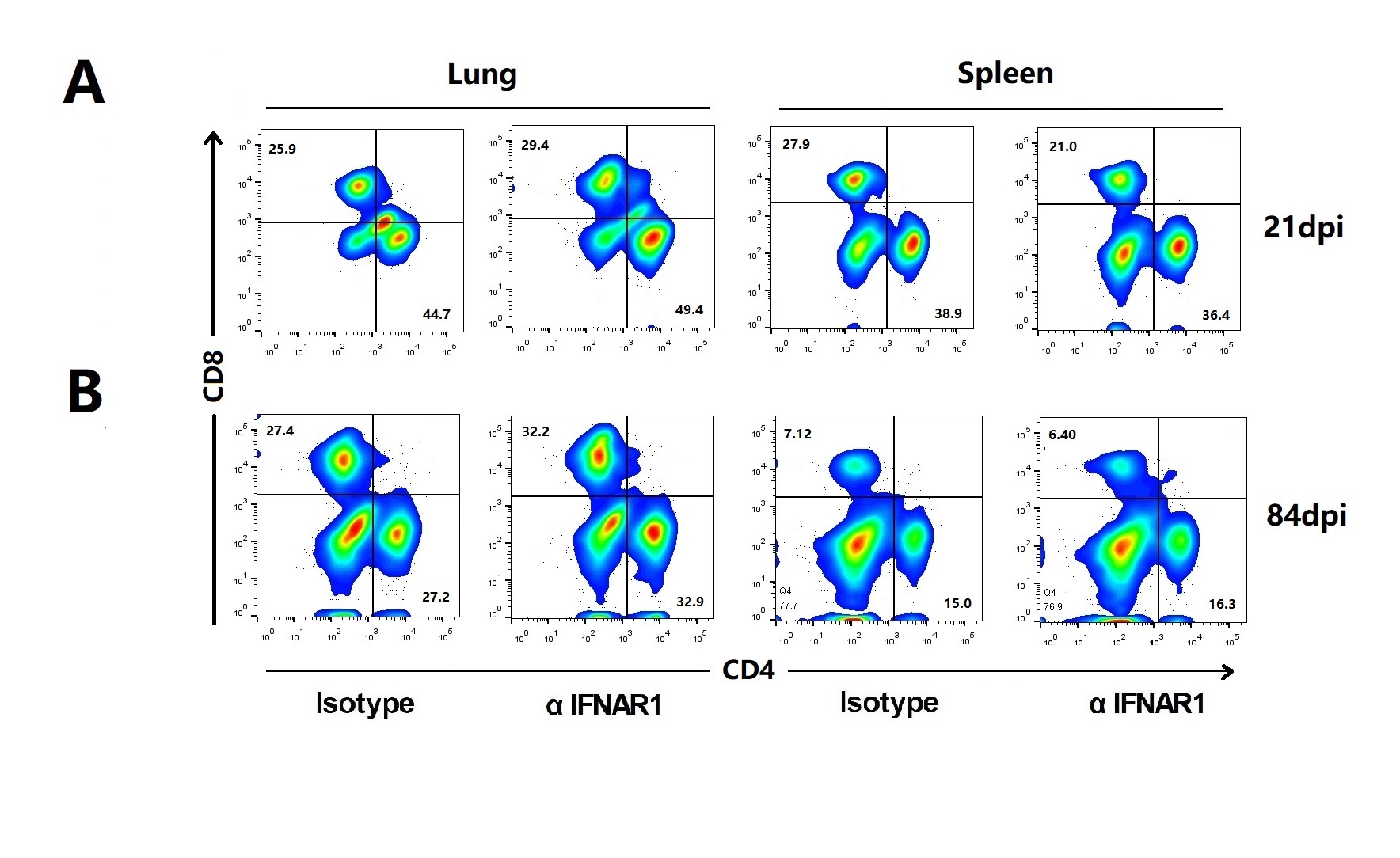

Supplement: Supplementary file 3 — Additional file 3: Figure S3. IFNAR1 blockade do not affect the population of CD4+ or CD8+ T cells in vivo. C57BL/6 mice were treated with αIFNAR1 or isotype antibodies one day prior to M. bovis infection. At 21and 84 days p.i, cell suspensions from lung and spleen were stimulated in vitro with ESAT-6:1–20 (10 μg/ml). (A-B) Representative flow cytometric plots of CD4+ or CD8+ T cells by ICS applied on total (right panel) lung cells and (left panel) splenocytes of mice at 21 and 84 days p.i (n = 3). Data is presented from two independent experiments. [file 12879_2019_4654_MOESM3_ESM.jpg]
